# CD4^+^ T cells apoptosis in *Plasmodium vivax* infection is mediated by activation of both intrinsic and extrinsic pathways

**DOI:** 10.1186/1475-2875-14-5

**Published:** 2015-01-05

**Authors:** Natália S Hojo-Souza, Dhelio B Pereira, Tiago AO Mendes, Lívia SA Passos, Ana Clara Gazzinelli-Guimarães, Pedro H Gazzinelli-Guimarães, Mauro S Tada, Graziela M Zanini, Daniella C Bartholomeu, Ricardo T Fujiwara, Lilian L Bueno

**Affiliations:** Department of Parasitology, Institute of Biological Science, Federal University of Minas Gerais, Av. Antônio Carlos 6627, 31270-901 Belo Horizonte, Minas Gerais Brazil; Research Centre in Tropical Medicine, Porto Velho, Rondônia Brazil; Clinical Research Institute Evandro Chagas, Oswaldo Cruz Foundation, Rio de Janeiro, Rio de Janeiro, Brazil

**Keywords:** Malaria, *Plasmodium vivax*, Apoptosis

## Abstract

**Background:**

Reduction in the number of circulating blood lymphocytes (lymphocytopaenia) has been reported during clinical episodes of malaria and is normalized after treatment with anti-malaria drugs. While this phenomenon is well established in malaria infection, the underlying mechanisms are still not fully elucidated. In the present study, the occurrence of apoptosis and its pathways in CD4^+^ T cells was investigated in naturally *Plasmodium vivax*-infected individuals from a Brazilian endemic area (Porto Velho – RO).

**Methods:**

Blood samples were collected from *P. vivax*-infected individuals and healthy donors. The apoptosis was characterized by cell staining with Annexin V/FITC and propidium iodide and the apoptosis-associated gene expression profile was carried out using RT^2^ Profiler PCR Array–Human Apoptosis. The plasma TNF level was determined by ELISA. The unpaired t-test or Mann–Whitney test was applied according to the data distribution.

**Results:**

*Plasmodium vivax*-infected individuals present low number of leukocytes and lymphocytes with a higher percentage of CD4^+^ T cells in early and/or late apoptosis. Increased gene expression was observed for TNFRSF1B and Bid, associated with a reduction of Bcl-2, in individuals with *P. vivax* malaria. Furthermore, these individuals showed increased plasma levels of TNF compared to malaria-naive donors.

**Conclusions:**

The results of the present study suggest that *P. vivax* infection induces apoptosis of CD4^+^ T cells mediated by two types of signaling: by activation of the TNFR1 death receptor (extrinsic pathway), which is amplified by Bid, and by decreased expression of the anti-apoptotic protein Bcl-2 (intrinsic pathway). The T lymphocytes apoptosis could reflect a strategy of immune evasion triggered by the parasite, enabling their persistence but also limiting the occurrence of immunopathology.

**Electronic supplementary material:**

The online version of this article (doi:10.1186/1475-2875-14-5) contains supplementary material, which is available to authorized users.

## Background

Malaria infection induces significant changes in the haematological parameters of the host. A decrease in the number of circulating blood lymphocytes (lymphocytopaenia) is a well-documented phenomenon in naturally infected individuals during acute *Plasmodium falciparum* [1–6] and *Plasmodium vivax* [5, 7–9].

Specific T cell depletion has been observed during infection with different species of *Plasmodium* (*Plasmodium chabaudi*, *Plasmodium vinckei* and *Plasmodium yoelii* YM) [10]. In human and experimental models of malaria, lymphocytopaenia has been attributed to the apoptosis of T lymphocytes and has been associated with high plasma levels of sIL-2R (soluble IL-2 receptor) [7, 11, 12] or the Fas/FasL system [4, 7, 13]; however, the mechanisms of apoptosis during malaria, particularly during *P. vivax* infection, is not fully elucidated. On the other hand, some studies have suggested that lymphocytopaenia during infection is caused by the reallocation of T cells at sites of inflammation, followed by reappearance of these cells in the blood during the treatment [3, 14, 15].

Considering the importance of CD4^+^ T cells in the protective immune response in vivax malaria, the objective of the present study was to verify possible mechanisms involved in lymphocytopaenia.

## Methods

### Study population

This study was performed with blood samples collected from 20 subjects naturally infected with *Plasmodium vivax* (*P. vivax*-infected donors) with uncomplicated symptomatic malaria, recruited at the Research Centre in Tropical Medicine (Porto Velho, Rondônia–Brazil), a malaria-endemic area. The control group consisted of 11 healthy donors (malaria-naive donors) from a non-endemic area (Belo Horizonte, Minas Gerais–Brazil) (Table 1). Parasitaemia was determined by well-trained microscopists from the Research Centre in Tropical Medicine. *Plasmodium vivax* mono-infection was confirmed by PCR, as previously described [16]. Haematological parameters were measured using an automated blood cell counter (ABX Pentra 90; Horiba Diagnostics, Kyoto, Japan) (Table 1). HIV, dengue and hepatitis testing was performed in all samples in order to exclude coinfections or comorbidities.

**Table 1:** **Demographic and haematological parameters of malaria-naive donors and** ***P. vivax*** **-infected donors (Mean ± SD)**

Parameters	Value for group		P value*
	Malaria-naive donors	***P. vivax***-infected donors	
	(n = 11)	(n = 20)	
Age (years)	34.0 (22–37)	38.5 (19–61)	
Gender			
Male	3	14	
Female	8	6	
Haemoglobin (g/dL)	14.05 ± 0.35	13.52 ± 0.29	0.263
Haematocrit	41.27 ± 0.90	38.47 ± 0.78	**0.028**
RBCs (cells/mm^3^)	4741000 ± 109300	4724000 ± 78940	0.901
WBCs (cells/mm^3^)	8118 ± 517.30	5475 ± 358.1	**<0.001**
Lymphocytes (cells/mm^3^)	2650 ± 221.5	1511 ± 122.5	**<0.0001**
Monocytes (cells/mm^3^)	177.1 ± 16.18	351.4 ± 34.49	**0.001**
Granulocytes	5270 ± 396.7	3686 ± 258.0	**0.002**
Eosinophils (cells/mm^3^)	179.5 ± 18.31	121.4 ± 9.91	**0.006**
Platelets (cells/mm^3^)	246500 ± 21290	124100 ± 13470	**<0.0001**

*Statistical differences determined by unpaired t-test.

### Ethics statement

This study was approved by the Ethics Committee of the Research Centre in Tropical Medicine (CAAEs: 0008.0.046.000-11, 0449.0.203.000-09), and written informed consent was obtained from all participants.

### Blood samples

Peripheral venous blood was collected in tubes containing EDTA and heparin. The blood collected with EDTA was used to confirm the diagnosis (*P. vivax* mono-infection) by PCR, to determine parasitaemia by microscopy (thick smears), to evaluate the haematological parameters and for cell phenotyping. Heparinized blood was used to obtain plasma for the cytokine assay and to obtain peripheral blood mononuclear cells (PBMCs) for the isolation of CD4^+^ T cells used to evaluate the apoptosis-associated gene expression profiles.

### Cell phenotyping

The apoptotic profile of the CD4^+^ lymphocyte population from both groups was characterized by Annexin V/FITC and propidium iodide (PI) cell staining (BD Biosciences, USA) using fresh whole blood. Briefly, the erythrocytes were lysed with ammonium chloride (150 mM) and washed twice in PBS. The cells were then stained with PerCP-conjugated monoclonal antibodies specific for CD4 (clone L200) (Becton Dickinson, USA) for 30 minutes in the dark at room temperature and later stained with Annexin V/FITC and PI. Phenotypic analyses were performed by flow cytometry with a FACScan flow cytometer (BD Biosciences, USA). Data were collected on 1x10^5^ lymphocytes (gated by forward and side scatter) and analysed using Flow Jo software (Tree Star Inc., USA).

### Apoptosis pathways

Blood samples from eight *P.vivax*-infected donors and three malaria-naive donors were used to evaluate the possible pathways involved in CD4^+^ T cells apoptosis. PBMCs were obtained from heparinized blood samples using a Ficoll density gradient centrifugation method (Histopaque, Sigma, USA). The blood sample was added gently above the Histopaque solution and then was centrifuged at 400 g for 40 minutes. Next, the phase containing the PBMCs was transferred to another tube, and the cells were washed twice with RPMI 1640 medium supplemented with 1% antibiotic (Gibco) and centrifuged at 400 g for 10 minutes. CD4^+^ T cells were isolated from PBMCs using a positive selection based by direct magnetic labelling using CD4 microBeads (Miltenyi Biotec, USA) following the manufacturer’s instructions. The enriched CD4^+^ T cells were resuspended in TRIzol Reagent (Life Technologies) and stored at – 80°C until further use. Cells were submitted to subsequent RNA isolation, which was performed following manufacturer’s instructions (Life Technologies, USA). The total RNA was treated with DNAse (Promega) and was then converted into cDNA using High-Capacity cDNA Reverse Transcription (Applied Biosystems) following the manufacturer’s instructions. PCR using primers for the human GAPDH gene was performed to confirm the cDNA synthesis.

The evaluation of apoptosis-associated gene expression profiles was carried out using RT^2^ Profiler PCR Array–Human Apoptosis (SABioscience), which determines 84 genes involved in different apoptosis pathways. The qPCR was performed using 30 ng of cDNA per well in an ABI7500 Real Time PCR System (Applied Biosystem, USA). The obtained data were analysed using the ΔΔCT method.

### Cytokine assay

The plasma TNF level was determined by enzyme-linked immunosorbent assay (ELISA) (R&D Systems) according to the manufacturer’s instructions. Biotin-labeled antibodies were used for detection, then revealed with streptavidin-HRP (Amersham Biosciences, USA) and the OPD substrate system (Sigma). The colorimetric reaction was read in an automated ELISA microplate reader at 492 nm. The cytokine concentration was calculated from the standard curve using seven-parameter curve fitting software (SOFTmaxH Pro 5.3, Molecular Devices, USA), and the results were expressed in pg/mL. The limit of detection for the assay was 15.6 pg/mL.

### Statistical analysis

Statistical analyses were carried out using Prism software 5.0 for Windows. Initially, the Kolmogorov-Smirnoff test was applied to verify whether the obtained data represent a normal distribution. The unpaired t-test or Mann–Whitney test was applied according to the data distribution. Grubb’s test was also used to verify outliers. A P-value < 0.05 was considered significant.

## Results

### *Plasmodium vivax*infection leads to leukopaenia, lymphocytopaenia and other haematological alterations

Previous reports have shown that malaria infection leads to alterations in haematological parameters during acute clinical episodes [5, 7–9]. Consistent with these data, the analysis of haematological parameters revealed that *P. vivax*-infected donors had a significant reduction in the number of leukocytes (p = 0.0002) and lymphocytes (p < 0.0001) compared with malaria-naive donors. Reductions in granulocytes (p = 0.002), eosinophils (p = 0.006), platelets (p < 0.0001) and haematocrit (p = 0.028), as well as an increase in monocytes (p = 0.001), were also observed (Table 1).

### *Plasmodium vivax*infection induces apoptosis of CD4^+^T cells

**Figure 1:**
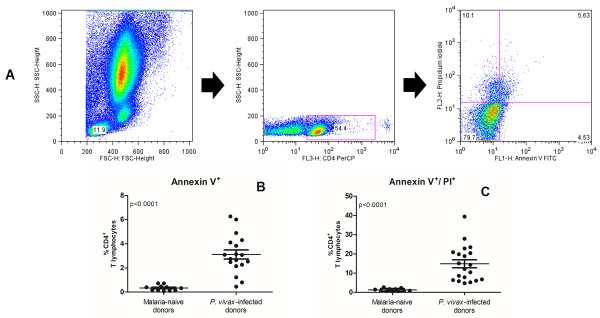
**Flow cytometric analysis of apoptosis.** Representative example of gating strategy used to characterize CD4^+^ T cells in apoptosis, using co-staining with Annexin-V and PI **(A)**. Percentage of CD4^+^ T cells in early **(B)** and late **(C)** apoptosis from malaria-naive donors (n = 11) and *P. vivax*-infected donors (n = 20). An unpaired t-test was used for comparison and the results were expressed as the mean ± SEM.

To determine whether the reduction in the number of lymphocytes was due to apoptosis of CD4^+^ T cells, whole blood samples of both *P. vivax*-infected donors and malaria-naive donors were stained with Annexin-V, PI and anti-CD4 antibody (Figure 1A). The results showed an increase in the percentage of CD4^+^ T cells in early apoptosis (Annexin V^+^) in *P. vivax*-infected donors (3.11%) compared to malaria-naive donors (0.34%) (p < 0.0001, Figure 1B). Analysis of CD4^+^ T cells also demonstrated that *P. vivax*-infected donors had a higher percentage of circulating cells in late apoptosis (Annexin V^+^/PI^+^) (14.86%) compared to malaria-naive donors (1.29%) (p < 0.0001, Figure 1C). Flow cytometric analysis of non-CD4 T cells and monocytes demonstrated a significant increase in the percentage of cells in early apoptosis (Annexin V^+^) in *P. vivax*-infected donors (2.03% and 1.93%, respectively) compared to malaria-naive donors (0.99% and 0.14%, respectively) (p < 0.0001 for both, see Additional file 1). No differences were observed in the frequency of non-CD4 T cells and monocytes in late apoptosis for *P. vivax* infected individuals (0.50% and 0.72%, respectively) when compared to malaria-naive donors (0.27% and 0.41%, respectively). On the other hand, further analysis focusing in granulocytes demonstrated a significant decrease of frequency of cells in late apoptosis in *P. vivax*-infected donors (1.99%) compared to malaria-naive donors (7.85%) (p = 0.0009 for both, see Additional file 1).

### Gene expression profiles indicate that *P. vivax*infection induces apoptosis in T CD4^+^cells by both extrinsic and intrinsic pathways

**Figure 2:**
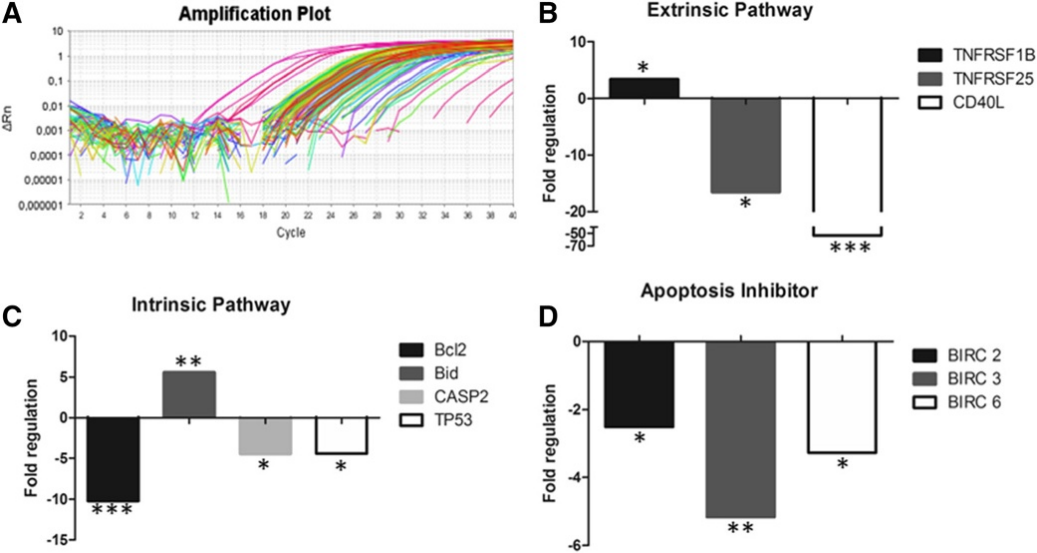
**Gene expression profiles associated with apoptosis pathways.** Representative example of amplification plot **(A)**. Apoptosis-associated gene expression was evaluated in malaria-naive donors (n = 3) and *P. vivax*-infected donors (n = 8). Genes were grouped according to the pathway they were involved in: extrinsic pathway **(B)**, intrinsic pathway **(C)** and apoptosis inhibitors **(D)**. Bars represent fold regulation of genes in CD4^+^ T cells from *P. vivax*-infected individuals in relation to the control group (malaria-naive donors) with statistically significant results. Statistical analyses were performed using PCR Array Data Analysis Web Portal (SABiosciences). *p < 0.05, **p < 0.01, ***p < 0.0001.

The observation that *P. vivax* infection induces apoptosis in CD4^+^ T cells prompted us to examine the possible pathways involved in this process. Thus, enriched CD4^+^ T cells (average 99.5%, see Additional file 2) were evaluated for apoptosis-associated gene expression profiles (Figure 2A).

Analysis of 84 genes involved in different pathways of apoptosis showed differential expression of 10 genes in *P. vivax*-infected patients in comparison to malaria-naive donors. These genes were separated into three groups based on extrinsic or intrinsic pathways and apoptosis inhibitors (Figure 2B, C and D, respectively). A significant increase was observed in the gene expression of TNFRSF1B (3.40; p = 0.026), an important death receptor expressed on the T cell surface. On the other hand, CD40L gene, which interacts with the CD40 receptor, and TNFRSF25, both of which are expressed on the cell surface, were down-regulated (p < 0.0001 and p = 0.028, respectively). Considering that the Bcl-2 family contains pro and anti-apoptotic members, different levels of expression (up- and down-regulation) were observed. While Bid expression was increased 5-fold in *P. vivax-*infected individuals (p = 0.005), Bcl-2 was 10-fold less expressed (p < 0.0001) in the same donors. The genes involved in PIDDosome pathway (tp53 and caspase-2) were significantly down-regulated in *P. vivax* malaria (p = 0.042 and p = 0.030; respectively). Some genes encoding inhibitors of apoptotic proteins (IAPs), also called Baculoviral inhibitors of apoptosis repeat containing (Birc), were also down-regulated.

### Plasma levels of TNF are higher in *P. vivax*-infected donors

**Figure 3:**
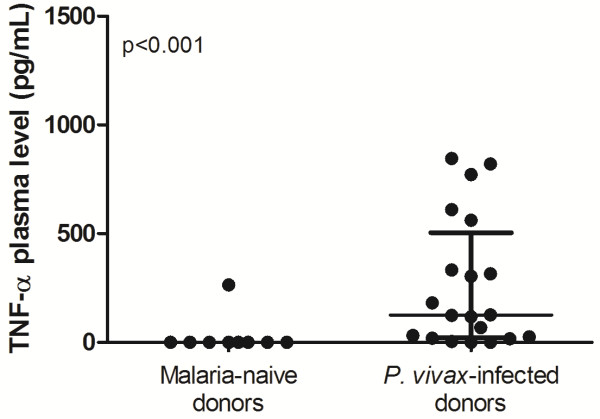
**Plasma level of TNF.** Levels of circulating TNF were determined by ELISA in malaria-naive donors (n = 11) and *P. vivax*-infected donors (n = 20). Mann–Whitney test was used for comparison and the result was expressed as the median with interquartile range.

After observing a higher expression of TNFRSF1 in individuals infected with *P. vivax*, the plasma levels of tumour necrosis factor (TNF) was determined. The results indicate that *P. vivax*-infected individuals had higher levels of plasma TNF compared to malaria-naive donors (p < 0.001, Figure 3), suggesting that *P. vivax*-infected individuals have more probability in activating the apoptosis cascade.

## Discussion

A better understanding in the apoptosis mechanisms induced by the *P. vivax* may be important to clarify the modulation of the human immune response. During vivax malaria, apoptosis is moslty found in CD4^+^ T cells with a minor contribution of monocytes and non-CD4 T cells, which present significant frequency of cells in early apoptosis. These data suggest that the leukopaenia and lymphocytopaenia observed in *P. vivax*-infected individuals is associated to apoptosis of CD4^+^ T cells. Moreover, *P. vivax*-infected individuals had higher levels of plasma TNF and increased gene expression of TNFRSF1B (death receptor) and Bid (proapoptotic protein), and decreased gene expression of Bcl-2 (anti-apoptotic protein).

Apoptosis is a cell death process tightly regulated that may be triggered by different stimuli. Extrinsic stimuli (TNF, FasL, TRAIL) can activate the apoptosis cascade through death receptor expressed on the cell surface, such as TNFR, Fas and TRAIL-R (extrinsic pathway) [17].

On the other hand, apoptosis can also be activated by intrinsic stimuli caused by oxidative stress, leading to alterations on mitochondrial membrane (intrinsic pathway) [18]. The activation of intrinsic pathway initiated by permeabilization of the outer mitochondrial membrane provoked by pro-apoptotic proteins members of Bcl family followed by the release of pro-apoptotic factors (cytochrome C, Smac/DIABLO, AIF) from the mitochondrial intermembrane space in the cytoplasm, resulting in downstream activation of caspase-9 [18, 19]. Regardless of the stimuli origin both pathways culminate in the activation of cysteine aspartyl protease 3 (caspase-3), caspase-6 and caspase-7, which results in chromatin condensation and DNA fragmentation.

Following the binding of TNF to its death receptor TNFR1, and subsequent recruitment of the TNF-associated death domain (TRADD) adapter protein, two events can occur: (i) activation of pro-inflammatory and potentially pro-tumor survival pathways through NF-κB, JNK and p38 or (ii) induction of apoptotic cell death via recruitment of Fas-associated death domain (FADD) and caspase-8 activation, culminating in the activation of caspase-3 [20, 21]. Thus, death receptor signaling, despite its name, can either induce cell death by apoptosis or activate non-apoptotic cell signaling pathways.

Although some studies have linked the occurrence of T lymphocyte apoptosis with high levels of soluble Fas ligand (sFasL) in human malaria, the involvement of the Fas/FasL system has not been observed in experimental models of malaria [10, 22]. Thus, unlike the studies that suggest the involvement of Fas/FasL in the lymphocytopaenia observed in patients with *P. falciparum* malaria, no increased expression of Fas and FasL genes in the CD4^+^ T lymphocytes of patients with *P. vivax* malaria was observed, similar to what had been found in malaria mouse models. In these experimental studies, apoptosis of *Plasmodium*-specific CD4^+^ T cells was associated with IFN-γ. In a mouse model of malaria using *P. berghei*, the parasite ortholog of macrophage migration inhibitory factor (PMIF) induced the upregulation of T-bet and IFN-γ and the downregulation of IL-7R, IL-7, IL-2 and Bcl-2 in T cells specific for the parasite [22]. These CD4^+^ T cells showed increased susceptibility to apoptosis and were associated with low expression of Bcl-2, but not with Fas/FasL signaling. Importantly, patients with *P. falciparum* cerebral malaria also had higher levels of serum PMIF compared with patients with uncomplicated malaria [22].

The results demonstrate that CD4^+^ T lymphocytes from patients infected with *P. vivax* malaria have increased expression of TNFR1 and Bid and decreased expression of anti-apoptotic Bcl-2 mRNA. Bcl-2 is an anti-apoptotic protein that exerts its action through interaction with pro-apoptotic proteins such as Bcl-2 associated X-protein (Bax) and Bcl-2 homologous antagonist killer (Bak), inhibiting their activity. Moreover, Bid is a pro-apoptotic protein that exerts its action directly activating Bax/Bak, culminating in cell apoptosis. Therefore, the reduction in the expression of Bcl-2 and the increase in the expression of Bid may lead to increased pro-apoptotic signaling in activated CD4^+^ T cells [23]. A signaling amplification loop is activated when caspase-8 levels are insufficient to activate caspase-3. Under these circumstances, caspase-8 cleaves Bid, yielding tBid (truncated Bid), which translocates to the mitochondria and directly activates Bax/Bad for pore formation, increasing the permeability of the mitochondrial outer membrane and culminating in apoptosis [20, 21, 23].

Apoptosis signaling pathways can be categorized into two types: (i) type I, mediated by TNFR1, with activation of caspase-3 by caspase-8 or by tBid, and (ii) type II, regulated by anti-apoptotic Bcl-2 proteins, which inhibit the function of pro-apoptotic proteins (such as Bax and Bad) to block apoptosis mediated by tBid or XIAP protein (inhibitor of caspase-9 activation – BIRC 4) [21]. Another important death receptor expressed on the cell surface is TNFRSF25 (also known as DR3), which is activated by TL1A [20].

The results showed that *P. vivax*-infected donors displayed decreased expression of TNFRSF25, suggesting that this gene is not involved in the apoptosis induced by *P. vivax*. Similar result was observed with CD40L, indicating no activation of CD40 signaling.

In addition to apoptosis activated by death receptors (extrinsic pathway) and mitochondria (intrinsic pathway), an alternative way to induce apoptosis has been proposed involving a protein called PIDD (p53-induced protein with death domain). This pathway, named PIDDosome, has been implicated in p53-dependent activation of caspase-2 in response to genotoxic stress [19]. The results showed a reduction in the gene expression of p53 and caspase-2 in malaria-infected individuals, suggesting that this pathway is not involved in *P. vivax*-induced apoptosis.

In the present study, it was observed a significant reduction in the gene expression of BIRC 2, 3 and 6 in *P. vivax*-infected donors, suggesting that these inhibitors of apoptotic proteins are not inhibiting the caspase pathway, thus contributing to apoptosis. The IAPs are an important group composed of eight proteins (BIRC 1–8) with anti-apoptotic roles involved in the caspase pathway. The BIRC family can be divided into two groups: BIRCs that act directly on caspases (BIRC 1–4, 7 and 8) and BIRCs that act in mitotic spindle formation (BIRC 5 and 6) [24]. On the other hand, pro-apoptotic molecules released from mitochondria (Smac/DIABLO and Omi/HtrA2) are capable of neutralize IAPs binding in BIRC 2, 3, 4, 5 or 6 through IAP-binding motif presents in the their N-terminal region [18].

Finally, it was detected an increase in the plasma levels of TNF in *P. vivax*-infected donors in comparison with malaria-naive donors. This finding is consistent with prior studies that have shown higher levels of TNF in the plasma of individuals infected by *P. vivax* [9, 25, 26] and suggest that *P. vivax*-infected individuals, which present with elevated plasma levels of TNF and increased expression of TNFR1, might likely have increased activation of the apoptosis cascade via the TNF pathway. Taken together, the results indicate that increased CD4^+^ T lymphocyte apoptosis during *P. vivax* malaria is mediated by either up-regulation of the TNFR1 pathway involving the Bid amplification loop (type I) and down-regulation of anti-apoptotic Bcl-2 signaling (type II). While T lymphocyte apoptosis might enable parasite persistence by limiting the occurrence of immunopathology, further studies are still required to determine whether this mechanism reflects a homeostatic host response against the presence of the parasite or whether it might reflect an immune evasion strategy triggered by the parasite.

## Electronic supplementary material

Additional file 1: **Flow cytometric analysis of apoptosis of CD4** ^**+**^ **T cells.** Percentage of non-CD4 T cells (A), monocytes (B) and granulocytes (C) in early and late apoptosis from malaria-naive donors and *P. vivax*-infected donors. Mann–Whitney test was used for comparison. Bars represent the median. (TIFF 356 KB)

Additional file 2: **Enrichment of T CD4** ^**+**^ **lymphocytes from PBMCs.** Representative example of gating strategy used to characterize CD4^+^ T cells after enrichment by magnetic activated cell sorting. In the left panel, flow cytometry pattern (FSC x SSC) of PBMCs. In the right panel, proportion of T CD4^+^ cell in regular PBMCs, after cell separation and in the CD4-depleted fraction. Data were collected on 1x10^5^ lymphocytes (gated by forward and side scatter) and analysed using Flow Jo software (Tree Star Inc., USA). (TIFF 169 KB)
