# Supplementary figures and images for: CD4+ T cells apoptosis in Plasmodium vivax infection is mediated by activation of both intrinsic and extrinsic pathways
Source: Malar J. 2015 Jan 5;14:5. doi: 10.1186/1475-2875-14-5 (PMC4326293; doi:10.1186/1475-2875-14-5)

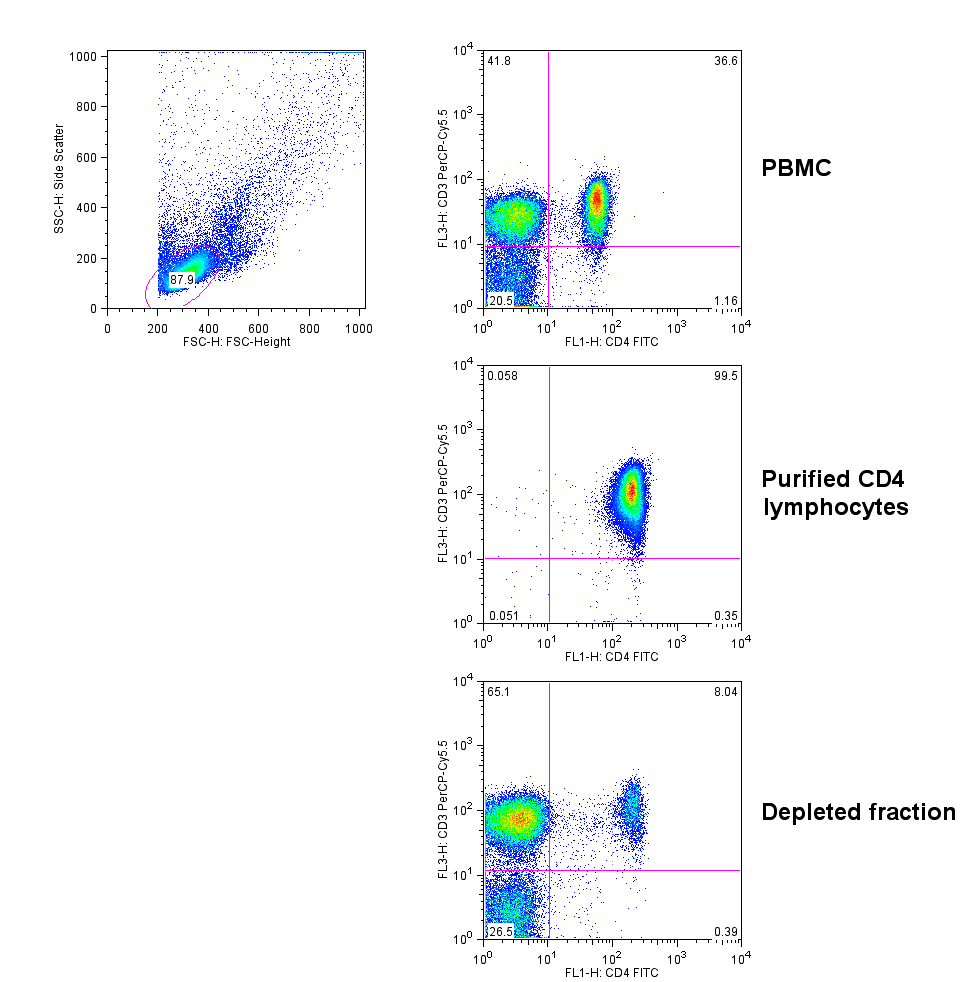

Supplement: Supplementary file 2 — Additional file 2: Enrichment of T CD4 + lymphocytes from PBMCs. Representative example of gating strategy used to characterize CD4+ T cells after enrichment by magnetic activated cell sorting. In the left panel, flow cytometry pattern (FSC x SSC) of PBMCs. In the right panel, proportion of T CD4+ cell in regular PBMCs, after cell separation and in the CD4-depleted fraction. Data were collected on 1x10^5^ lymphocytes (gated by forward and side scatter) and analysed using Flow Jo software (Tree Star Inc., USA). (TIFF 169 KB) [file 12936_2014_3674_MOESM2_ESM.tiff]
